# Sequential codend improves quality of trawl-caught cod

**DOI:** 10.1371/journal.pone.0204328

**Published:** 2018-10-10

**Authors:** Jesse Brinkhof, Stein H. Olsen, Ólafur A. Ingólfsson, Bent Herrmann, Roger B. Larsen

**Affiliations:** 1 Norwegian College of Fishery and Aquatic Science, University of Tromsø, Tromsø, Norway; 2 Nofima AS, Tromsø, Norway; 3 Institute of Marine Research, Bergen, Norway; 4 SINTEF Ocean, Hirtshals, Denmark; Fred Hutchinson Cancer Research Center, UNITED STATES

## Abstract

Trawl-caught fish are frequently associated with deteriorated catch quality. This study presents a new dual sequential codend concept with the aim of improving the quality of trawl-caught fish by minimizing the frequency and severity of catch damage. During towing, the fish are retained in an anterior codend segment with the legislated mesh size. A quality improving codend segment, is attached to the aft part of the first codend segment. Its entrance is closed during the towing phase and opened at a predefined depth during haul-back. Comparing the quality of cod (*Gadus morhua* L.) retained in the sequential codend with cod caught in a conventional codend, demonstrated a significant improvement in the catch quality, i.e. reduction in catch damages. Cod caught in a conventional codend had only a 3.6% probability of being without visually detectable catch damage. The probability for catching cod without catch damage was five times higher when using the dual sequential codend. Furthermore, cod caught in the sequential codend had a significantly reduced probability of incurring specific catch damage, such as gear marks, poor exsanguination, ecchymosis, and skin abrasions.

## Introduction

Fish caught with trawls are frequently associated with reduced quality compared with those caught using other fishing methods [1–4]. From a fisheries perspective, poor catch quality may imply reduced price per quantity caught, and, thus, reduced economic yield. This is mainly because poor catch quality limits the applications of fish for various products, as well as reduces their shelf life [5–6]. From a management perspective, poor catch quality is believed to increase the risk of discarding and high-grading [7], thus contributing to unaccounted fishing mortality. Hence, poor catch quality is not in accordance with sustainable resource exploitation, from neither a fishery nor management point of view. As deteriorated catch quality due to the catching process is impossible to improve a posteriori, even with best practice processing procedures, preventing catch damage is key to improve catch quality.

The most common visually detectable injuries and quality defects encountered among trawl-caught cod are skin abrasions, gear marks, internal and external ecchymosis, pressure injuries, and insufficient exsanguination [4, 8–10]. The rate and severity of the injuries and quality defects is likely to be affected by various factors such as towing time and catch sizes [9, 11], as well as seasonal and spatial variation [8, 12–14]. The catching method and the handling of the catch are two important factors in determining the final quality [3, 15]. During trawling, a crucial phase that greatly affects catch quality is haul-back. During haul-back, the fish are densely packed in the codend, making swimming difficult or impossible, especially for those located in the aft of the codend. Such dense crowding prevents fish from moving their operculum and thus reduce or inhibit water flow over the gill arches, resulting in hypoxic and anoxic conditions, with subsequent suffocation. Furthermore, it is believed that the coarse netting with large knots often used in demersal trawl codends also causes skin abrasions, gear marks and ecchymosis. When the codend is hauled up the stern, the fish are exposed to the crushing pressure of the surrounding catch, especially in the far end of the codend. This is believed to contribute extensively to the level of gear marks, skin abrasions, internal and external ecchymosis as well as pressure injuries on the fish.

This study aimed to improve the quality of trawl-caught fish by changing codend design used in the Barents Sea bottom trawl fishery for Northeast Atlantic cod (*Gadus morhua* L.). The fishery for cod is the most important fishery in the Barents Sea [16]. Approximately 70% of the annual Northeast Atlantic cod quota is caught using bottom trawls [17]. Although the fishery amelioration has mainly focused on catch efficiency and catch quantity, there has been a growing focus in recent years on improving catch quality (pers. comm., 1^st^ and 2^nd^ author). Codend design is believed to be vital for achieving good-quality catches. However, trawl configuration, including the codend, is regulated by law to ensure the release of undersized fish. Most undersized fish are supposed to be released by compulsory size-selective grid sections. The most-applied grid system is the Flexigrid; however, because this grid system does not release all undersized fish [18], the legislation requires that the codend also has size selective attributes. Therefore, the fish must be collected in a conventional codend that maintains the mesh size legally required during the towing phase. Hence, the new concept comprises a dual sequential codend, in which the first codend segment fulfils the size-selective properties required followed by a second quality improving codend segment. The purposes of the design of the quality-improving codend segment were to reduce the water flow inside the codend, avoiding the fish being packed too densely. Also, compared to the coarse netting in conventional codends, the design was supposed to reduce the mechanical strain on the catch. Furthermore, the codend was designed to hold as much water as possible when hauled up the stern of the trawler so that the fish were kept for as long as possible in water to minimize the risk of pressure-induced catch injuries. Hence, this codend was designed to reduce the amount and severity of gear marks, skin abrasions, ecchymosis, and pressure injuries often seen on trawl-caught fish, and, in this way, improve fish quality.

The main objective of this study is to quantify and compare catch damage on fish from a conventional codend and the sequential codend. Specifically, the aim of this study was to investigate the following research objectives:

- Document the catch damage on cod caught with the conventional codend, and the sequential codend, and compare the amount and severity of catch damages between the two codends.
- Document the functionality of the new sequential codend concept.

## Material and methods

### Dual sequential codend concept

**Fig 1:**
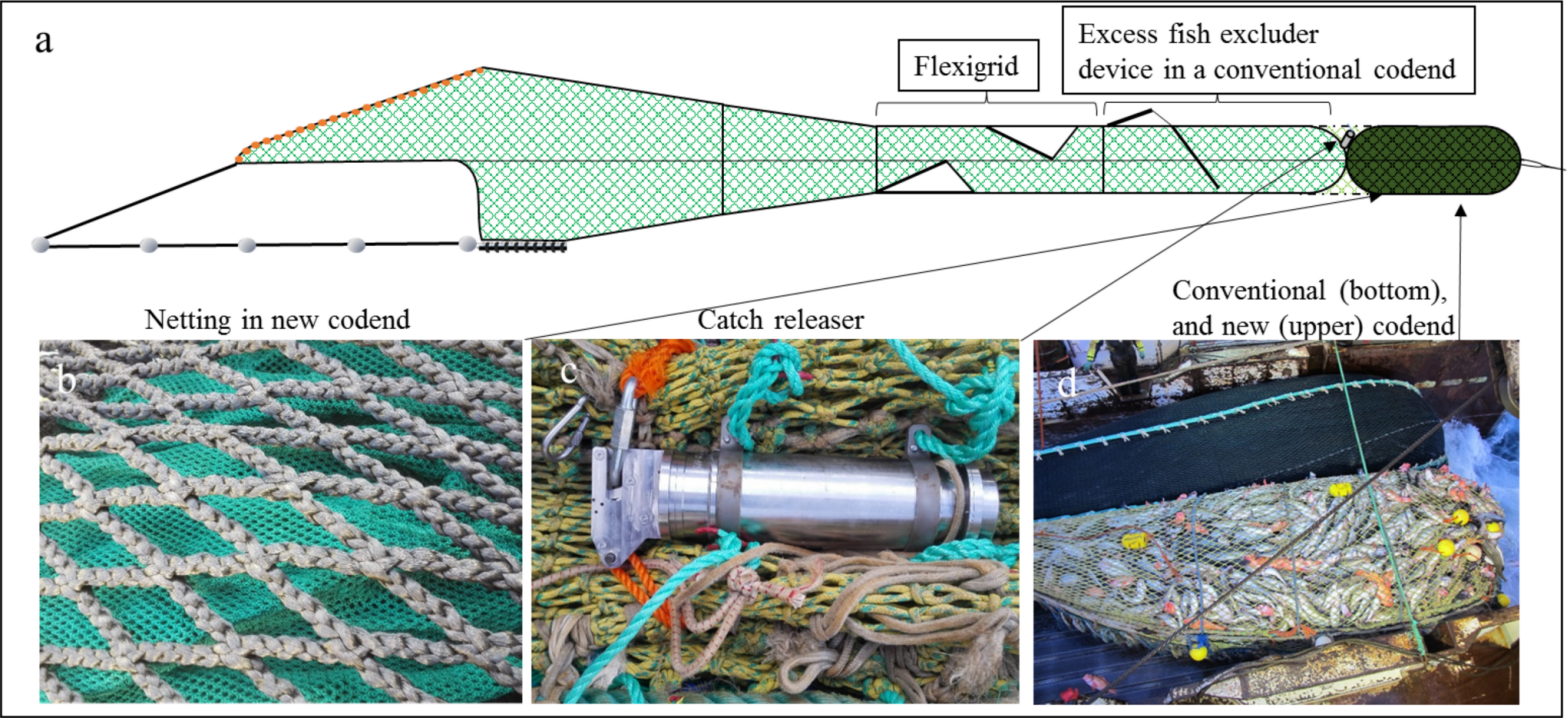
The setup of the trawls (a), the aft part shows the section with a Flexigrid, followed by an excess fish excluder device in the first codend segment, equivalent to a conventional codend. A second codend segment with quality-improving attributes was attached after the size-selective codend segment and kept closed during fishing with the catch releaser. (b) The netting in the quality-conserving codend segment. (c) The catch release mechanism. (d) The conventional codend trawl beside the trawl with the quality-conserving codend.

The new dual sequential codend concept was designed with the purpose of improving catch quality. The concept comprised two codends segments. The first codend segment was similar to a conventional codend, having the legislated mesh size, and required selective attributes. The codend segment comprised single-braided, Ø8-mm twine polyethylene netting, with a mesh size of 130.4 (± 2.4) mm, and a total length of 6.7 m. The second codend segment was attached to the end of the size-selective codend segment (Fig 1A). The second segment was 10 m long and comprised four panels. It had a nominal mesh size of 6.0 mm, and a circumference of 1440 meshes (360-meshes wide in each panel). To ensure its strength, the small meshed netting had an outer codend of knotless Ultra cross with a nominal mesh size of 112 mm (90 meshes in circumference) (Fig 1B). The four lastridge ropes were 5% shorter than the codend netting.

The entrance of the second codend segment with quality-improving attributes was closed when fishing and opened during haul-back with a hydrostatic codend releaser (www.fosstech.no, Fig 1A and 1C). The codend entrance was closed with a choking rope, attached to the releaser. The catch releaser had a pressure accumulator that was charged by ambient pressure during descent. During haul-back, and once fishing was ended, the accumulated pressure was used to open a release hook at a depth pre-set to 110 m. When the choking rope is detached, the passage between the two codend segments is opened, enabling fish to pass into the posterior quality-improving codend segment (Fig 1C and 1D).

**Fig 2:**
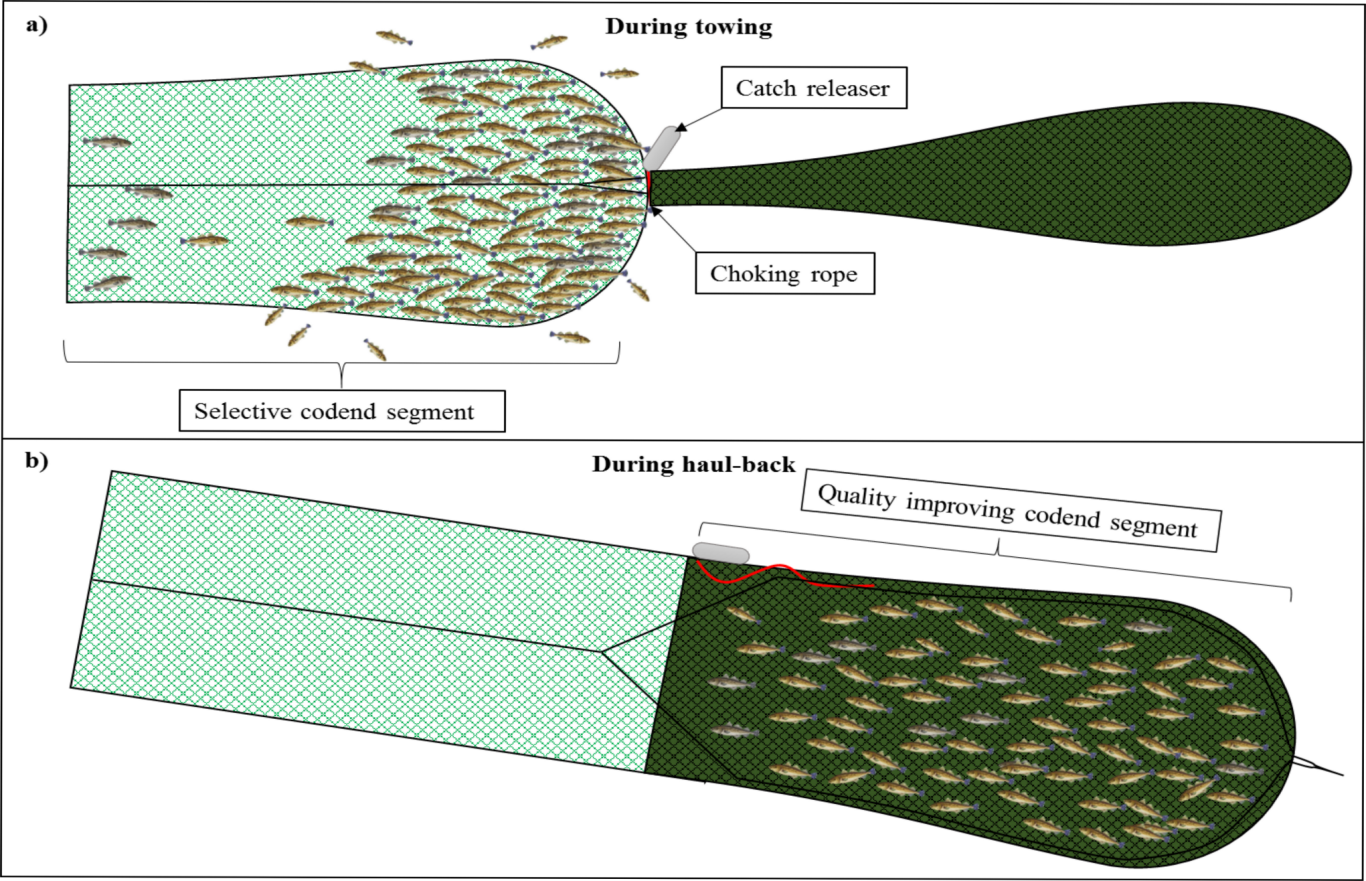
The dual sequential codend concept showing the first codend segment (a), where the fish are retained during towing, with the selective properties as legislated, followed by the quality-improving codend segment (b), in which the catch falls back during haul-back. The grey cylinder represents the catch releaser with the choking rope (red).

Hence, the new concept comprises a dual sequential codend, in which the first codend segment fulfils the size-selective properties required by law. The second codend segment, which entrance is closed during towing along the seabed (Fig 2A), is designed to improve catch quality as well as avoid size selection during haul-back and at the surface (Fig 2B).

### Sea trials and trawl rigging

The assessment of catch damages on cod, caught in the conventional codend and the sequential quality-improving codend, was performed onboard the commercial trawler M/Tr ‘J. Bergvoll’ (57.3 m, 3900 HP), from 7 to 19 April 2017. The sea trials were carried out in the Barents Sea on the fishing grounds north of Norway (N 71°08′–71°18′; E 28°33′–26°09′). Two identical Alfredo 5 trawls were applied as twin-trawls, enabling a direct comparison between the two codend setups. This paired comparison reduced uncontrolled variance in the data encountered in an alternate haul setup because of varying fishing conditions. The trawls were 530 meshes in circumference with a mesh size of 155 mm. The headlines of the trawls were 38 m long and had 25 m long extension bridles. The fishing lines were 21 m long, with 21 m long rockhopper gear with Ø61-cm rubber discs. The sweeps were 72 m long, the outer ones connected to a set of Thyborøn VF14 semi-pelagic otter boards (each 9 m^2^, weighing 4400 kg), whereas the inner sweeps were connected to a 5000 kg roller clump. Both trawls were equipped with a flexi-grid mounted in a two-panel section (Fig 2A) [18]. The flexi-grids, which are one of the mandatory selective devices in this fishery, had a bar spacing of 55 mm. The dual sequential codend with the catch releaser was attached to the starboard trawl. The port trawl was equipped with a conventional codend. Both the trawl gear as well as rigging is commonly applied on board commercial factory trawlers in the Barents Sea trawl fishery. To avoid excessively large catches, and to catch approximately the same catch sizes in each haul, an excess fish excluder device was inserted between the codend and the grid section (Fig 2A). Such excess fish excluder devices are frequently used by commercial trawlers when the densities of fish are high.” The excess fish excluder comprises a 3.2 m long panel, termed a fish lock, which is made of 80 mm mesh netting. The panel is obliquely sewn to the section with an opening between the aft part of the panel and the lower trawl panel. The fish lock prevents fish that have entered the codend from swimming forwards out of the codend. A rectangular hole (0.65 × 0.80 m) is cut in the upper panel of the trawl, 0.5 m in front of the fish lock, and covered with a rubber mat (1.0 × 1.5 m). The anterior part of the rubber mat is sewn to the trawl, whereas the aft part is kept down by three 0.6 m long rubber bands. The rubber mat closes the hole during fishing and is lifted upwards when catch accumulates because of the opening of the meshes and alternating water current, thus releasing any excess fish caught. Additionally, a set of catch sensors (MARPORT) was used for assessing the amount of catch during trawling. The trawl geometry, otter board spread, trawl symmetry, trawl height, and bottom contact) were monitored using sensors from SCANMAR.

### Data sampling

The data was sampled onboard a trawler during commercial fishing operations. Immediately after hauling the catch onboard, 25 cod were randomly sampled from each codend on deck. The codends were emptied into the holding bins, carefully, and one at a time. To ensure random sampling, approximately 1/3^rd^ of the fish were collected at the end of the codend, 1/3^rd^ from the middle, and 1/3^rd^ from the beginning of the codend. The fish sampled from the codends on deck were not exposed to any strain in addition to what all fish experience during commercial fishing. The sampled fish from both codends were stunned by a blow to the head, and then killed and exsanguinated in separate tanks containing 800 l of running seawater (ca. 50 l.min^-1^) for 30 min. The exsanguination time was equal to the time commonly practiced in the fishing industry. After 30 min, the water was drained from the tanks and the fish were evaluated using a Catch Damage Index for catch defects inflicted during the catching process (Table 1) [1, 4, 19]. To increase accuracy a four-level Catch Damage Index was chosen [19], rather than a three-level [1,4]. The assessment was performed as a blinded experiment by professionally trained personnel. The order of the assessment of catch damages from the two codends was randomly alternated to avoid any potential bias. Since the fish were not exposed to any additional strain compared to commercial fishing, and the data sampling was conducted on dead fish, no special specific permits were required, and the experiment did not cause any concerns regarding animal welfare.

**Table 1:** Catch Damage Index used for evaluating the damage inflicted on fish during trawling.

Catch damage	Score							
	Flawless	Slightly	Moderate	Severe	Description			
**Poor exsanguination**	0	1	2	3	Improper bleeding, blood in veins			
**Ecchymosis**	0	1	2	3	Discoloration of skin, bruises			
**Gear marks**	0	1	2	3	Marks on skin caused by gear contact			
**Pressure injuries**	0	1	2	3	Injuries caused by crushing			
**Skin abrasions**	0	1	2	3	Loss of scales			

### Data analysis

The difference in the probability of cod obtaining a specific damage score between the catches caught with the conventional codend and the sequential codend was investigated based on the collected samples. According to the Catch Damage Index, five different categories were looked at, ‘poor exsanguination’, ‘ecchymosis’, ‘gear marks’, ‘pressure injuries’ and ‘skin abrasions’ (Table 1). For each category, the severity of damage was graded applying scores from 0 to 3 / a score of 0, 1, 2, or 3 (Table 1). External catch related damages have a direct impact on the applicability for various fish products, as well as they are interrelated with internal damages as well as stress levels.

For fish caught in the conventional or the dual sequential codend, the expected average value $\hat{p_{as}}$ for the probability for a score *s* on category *a* was determined using Eq 1:

$$\begin{array}{c} \hat{p_{as}}=\frac{\sum_{j=1}^{m}\left\{\frac{1}{n_{j}}\sum_{t=1}^{n_{j}}equal(s,k_{ajt})\right\}}{m}\\ with\\ equal(s,k)=\left\{ \begin{array}{c} 1\forall k=s\\ 0\forall k\neq s \end{array}\right. \end{array},$$ (1)

where *m* is the number of hauls conducted, *n*_*j*_ is the number of fish given a score in haul *j*, and *k*_*ajt*_ is the score given on category *a* to fish *t* evaluated in haul *j*.

The probability $\hat{{pm}_{as}}$ of obtaining a score that does not exceed *s* on category *a* (i.e. the probability of obtaining a given score or lower), was investigated using Eq 2:

$$\begin{array}{c} \hat{{pm}_{as}}=\frac{\sum_{j=1}^{m}\left\{\frac{1}{n_{j}}\sum_{t=1}^{n_{j}}lequal(s,k_{ajt})\right\}}{m}\\ with\\ lequal(s,k)=\left\{ \begin{array}{c} 1\forall k\leq s\\ 0\forall k>s \end{array}\right. \end{array}$$ (2)

Eqs 1 and 2 provide an evaluation of each category separately. However, it is also of interest to investigate the probability of a fish scoring *s* or maximum *s* on two or more of the categories simultaneously. The combined score is relevant as in many cases it is the total amount of damages that results in good or bad quality and possible downgrading. To estimate such probabilities, Eqs 1 and 2 were extended to all possible combinations of the categories to Eqs 3 (3.1, 3.2, 3.3 and 3.4) and 4 (4.1, 4.2, 4.3, 4.4), respectively:

$$\hat{p_{as}p_{bs}}=\frac{\sum_{j=1}^{m}\left\{\frac{1}{n_{j}}\sum_{t=1}^{n_{j}}equal(s,k_{ajt})\times equal(s,k_{bjt})\right\}}{m}$$ (3.1)

$$\hat{p_{as}{p_{bs}p}_{cs}}=\frac{\sum_{j=1}^{m}\left\{\frac{1}{n_{j}}\sum_{t=1}^{n_{j}}equal(s,k_{ajt})\times equal(s,k_{bjt})\times equal\left(s,k_{cjt}\right)\right\}}{m}$$ (3.2)

$$\hat{p_{as}{{p_{bs}p}_{cs}p}_{ds}}=\frac{\sum_{j=1}^{m}\left\{\frac{1}{n_{j}}\sum_{t=1}^{n_{j}}equal(s,k_{ajt})\times equal(s,k_{bjt})\times equal\left(s,k_{cjt}\right)\times equal\left(s,k_{djt}\right)\right\}}{m}$$ (3.3)

$$\hat{p_{as}{{p_{bs}p}_{cs}p}_{ds}p_{es}}=\frac{\sum_{j=1}^{m}\left\{\frac{1}{n_{j}}\sum_{t=1}^{n_{j}}equal(s,k_{ajt})\times equal(s,k_{bjt})\times equal\left(s,k_{cjt}\right)\times equal\left(s,k_{djt}\right)\times equal\left(s,k_{ejt}\right)\right\}}{m}$$ (3.4)

And

$$\hat{{pm}_{as}{pm}_{bs}}=\frac{\sum_{j=1}^{m}\left\{\frac{1}{n_{j}}\sum_{t=1}^{n_{j}}lequal(s,k_{ajt})\times lequal(s,k_{bjt})\right\}}{m}$$ (4.1)

$$\hat{{pm}_{as}{{pm}_{bs}pm}_{cs}}=\frac{\sum_{j=1}^{m}\left\{\frac{1}{n_{j}}\sum_{t=1}^{n_{j}}lequal(s,k_{ajt})\times lequal(s,k_{bjt})\times lequal\left(s,k_{cjt}\right)\right\}}{m}$$ (4.2)

$$\hat{{pm}_{as}{{{pm}_{bs}pm}_{cs}pm}_{ds}}=\frac{\sum_{j=1}^{m}\left\{\frac{1}{n_{j}}\sum_{t=1}^{n_{j}}lequal(s,k_{ajt})\times lequal(s,k_{bjt})\times lequal(s,k_{cjt})\times lequal\left(s,k_{djt}\right)\right\}}{m}$$ (4.3)

$$\hat{{pm}_{as}{{{pm}_{bs}pm}_{cs}pm}_{ds}{pm}_{es}}=\frac{\sum_{j=1}^{m}\left\{\frac{1}{n_{j}}\sum_{t=1}^{n_{j}}lequal(s,k_{ajt})\times lequal(s,k_{bjt})\times lequal\left(s,k_{cjt}\right)\times lequal\left(s,k_{djt}\right)\times \times lequal\left(s,k_{ejt}\right)\right\}}{m}$$ (4.4)

Estimation of the uncertainties in the expected values for the probability parameters calculated based on Eqs 1–4 needed to consider several aspects: (i) the average score might vary between hauls because of uncontrolled effects in the fishing process; (ii) the average score for the individual hauls is subject to within haul-variability because a limited sample of fish was evaluated from each haul; and (iii) there might be correlations between the probabilities of the scores between categories, which complicates the estimations of uncertainties for the combined probabilities from Eqs 3 and 4. To account correctly for the aforementioned uncertainties in the estimations, a double bootstrap method was adapted, which is well established for evaluating fishing gear selectivity and catch efficiency for trawl fisheries, which are known to be subject to similar structures of uncertainty [19–23]. The procedure accounted for between-haul variation in the obtained scores by selecting *m* hauls with the replacement from the pool of hauls during each bootstrap repetition. Within-haul uncertainty in the obtained scores was accounted for by randomly selecting fish with replacement from the selected haul. The number of fish selected from each haul was the same as the number of fish evaluated for that haul (*n*_*j*_). The resulting data for each bootstrap were then used to estimate the expected category probabilities based on Eqs 1–4. The use of bootstrapping, that simply resamples the experimental data, does not require any assumptions regarding correlation or not between scores for different categories, making the estimation of uncertainties for the combined probabilities straightforward. In total, 1000 bootstrap repetitions were performed and the Efron 95% percentile confidence limits were calculated [24] for the estimated values.

The difference *Δr* in average probability for fish quality scores between fishing with the conventional and the dual sequential codend for an arbitrary parameter *r* obtained from Eqs 1–4 was obtained based on the parameter value obtained for each of the two codend types (Eq 5):

$$\Delta r=r_{QIC}-r_{CC}$$ (5)

where subscripts CC and QIC denote the Conventional Codend and the Quality Improving Codend, respectively. By taking advantage of the two codends being fished simultaneously in the same fishing hauls because of the paired experimental setup, they will be at least partly subject to the same between-haul variation potentially affecting the values of *r*. This was used to improve the precision in the estimation of *Δr* in terms of the 95% confidence intervals (CIs) by simultaneously estimating *r_QIC_, r_CC_* and *Δr* based on Eq 5 in the same bootstrap repetitions. This resulted in a bootstrap population of results for *Δr*, from which the Efron 95% CIs were calculated. For cases where the CI for Δ*r* did not contain 0.0, the sequential codend was deemed to have a significant effect on the value of parameter *r*. A positive value implied an increased score probability for the sequential codend, whereas a negative value implied a reduced score probability for cod caught in the sequential codend compared with the fish caught in the traditional codend.

The estimation procedures described earlier were implemented in the analysis tool SELNET [25]. The results were exported for graphical presentation in R [26].

## Results

**Fig 3:**
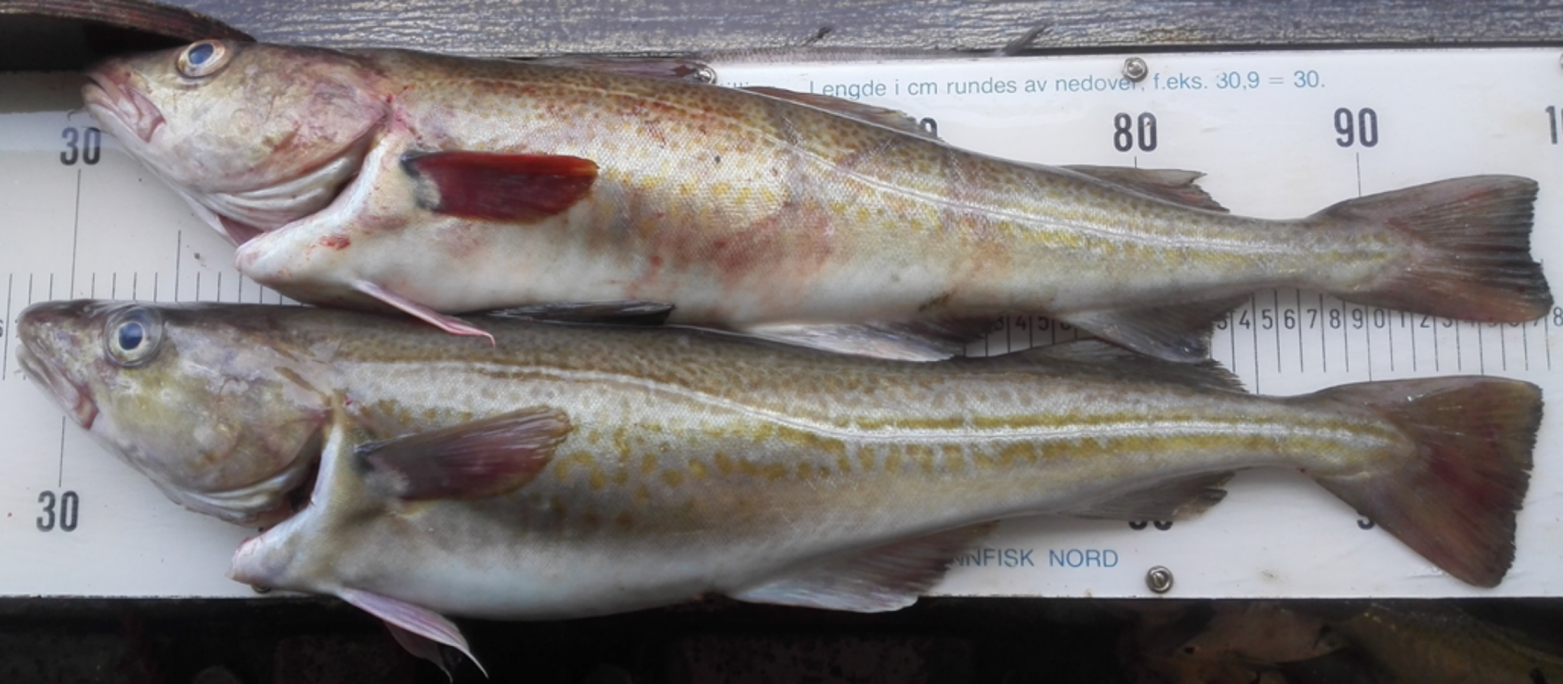
An example of a good-quality cod (b) that would score 0 for all five categories within the Catch Damage Index. The cod in (a) is an example of a poor-quality cod, obtaining a score 2 on ‘gear marks’, 3 on ‘ecchymosis’, and 1 on ‘skin abrasion’.

A total of 16 hauls were conducted with the dual sequential codend and a conventional codend in a twin-trawl setup. Six hauls were considered invalid because of broken gear, and other events that could influence the catch quality and size selection, such as buffer towing [19, 27]. None of the invalid hauls were caused due the modified codend. During the experiments no dead fish were observed in the codend catches when hauled on board, i.e. all sampled fish were alive. From each of the ten valid hauls, 25 cod were randomly sampled from each codend for the assessment of the catch damage, resulting in a total of 500 cod evaluated for catch defects. The two cods presented in Fig 3 are typical examples of a good-quality cod (lower), obtaining score 0 for all categories within the Catch Damage Index, and a poor-quality cod (upper), obtaining score 2 for ‘gear marks’, 3 for ‘ecchymosis’, and 1 for ‘skin abrasion’.

Position, depth, haul duration and the total catch weight in each codend were recorded (Table 2). For hauls 5 and 10, the catch weight in the respective codends were estimated based on the total catch weight, because the catches were not kept separate during processing. The mean fish length, presented in Table 2, demonstrate that the average fish length caught in the two codends were similar.

**Table 2:** Towing depth, start time, haul duration, catch weight, and mean fish length in the conventional and sequential codends.

Haul number	Depth (m)	Haul duration (hh:mm)	Catch regular codend (kg)	Catch sequential codend (kg)	Fish length (cm) regular codend (Mean ± SD)	Fish length (cm) sequential codend (Mean ± SD)
**1**	250	05:55	6156	5578	67.2 ± 6.82	70.6 ± 8.11
**2**	250	04:40	9469	8257	64.9 ± 4.55	66.7 ± 8.63
**3**	220	03:17	7489	7350	70.2 ± 6.69	66.3 ± 8.80
**4**	220	05:03	8849	8664	69.2 ± 6.63	70.3 ± 8.79
**5**	210	03:14	6778*	6778*	65.9 ± 8.69	70.8 ± 13.39
**6**	210	05:35	6922	5873	66.3 ± 7.75	69.3 ± 8.04
**7**	250	03:42	4057	7098	65.9 ± 7.03	71.3 ± 6.75
**8**	270	03:49	4348	4918	65.8 ± 9.53	75.3 ± 8.64
**9**	220	02:36	2633	1011	70.6 ± 7.47	71.1 ± 8.4
**10**	250	04:19	4500*	2700*	66.1 ± 3.41	68.9 ± 6.19

*The catches from hauls 5 and 10 were not separated accurately during processing, therefore the catch volume is estimated.

**Fig 4:**
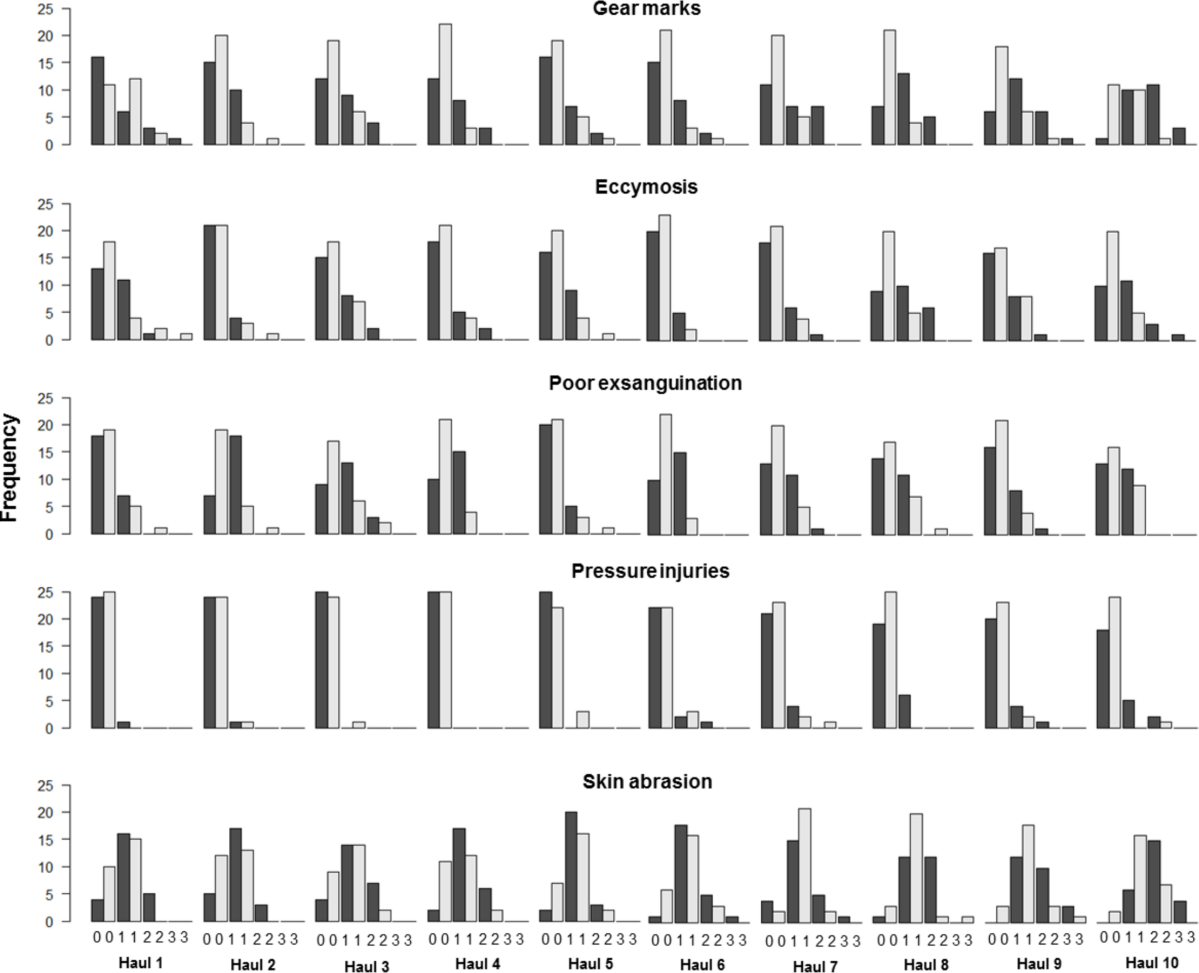
The score frequency for the five damage categories. Catches from the conventional (black) and sequential (grey) codends.

Score 0, for the categories ‘gear marks’, ‘ecchymosis’, ‘poor exsanguination’, and ‘skin abrasion’ occurred more frequently in the catches caught with the sequential quality-improving codend compared with the catches from the conventional codend (Fig 4). By contrast, scores 1, 2, and 3 occurred more frequently in the conventional codend catches compared with the sequential codend (Fig 4). The same pattern was seen for the category ‘pressure injuries’, although the discrepancy was lower in magnitude compared with the other four categories. The observed discrepancies in the frequency distribution of the scores between the codends illustrate the improved quality of fish retained in the sequential codend (Fig 4).

The improvement in fish quality was significant where the CIs from the difference in probabilities (Eq 5) did not contain 0.0. The discrepancies between the estimated probabilities for obtaining a given score for cod are presented in Table 3. Cod caught in the quality-improving sequential codend had a 14%–32% increased probability of obtaining score 0 for all categories, except ‘pressure injuries’, depending on the damage category or category combination (Table 3). Specifically, the probability of obtaining score 0 for cod caught in the sequential codend increased by 28% for ‘gear marks’, 18% for ‘ecchymosis’, and 26% for ‘poor exsanguination’. The probability of obtaining scores 0 and ≤1 for all five categories increased by 16% and 22% with the new codend concept, respectively, whereas the probability of obtaining score 2 was reduced by 20%. In addition, the probability of obtaining score 2 was reduced for ‘gears marks’ (13%). The probability of obtaining score 1 for ‘ecchymosis’ and ‘poor exsanguination’ was reduced by 12 and 26%, respectively, with the sequential concept (Table 3).

**Table 3:** Increased probability for obtaining a given score for all cases investigated, with 95% confidence intervals (CIs) in parenthesis.

Category	Improvement in score probability (95% CIs)					
	= 0	= 1	= 2	= 3	≤ 1	≤ 2
Gear marks	**0.28 (0.11–0.40)**	−0.12 (−0.23–0.00)	−**0.13 (**−**0.22**–**-0.05)**	−0.02 (−0.06–0.00)	**0.16 (0.06**–**0.26)**	0.02 (0.00–0.06)
Ecchymosis	**0.18 (0.07–0.29)**	−**0.12 (0.22**–**0.03)**	−0.05 (−0.12–0.00)	0.00 (−0.02–0.02)	**0.05 (-0.01**–**0.12)**	0.00 (−0.02–0.02)
Poor exsanguination	**0.26 (0.14–0.38)**	−**0.26 (**−**0.38**–−**0.14)**	0.00 (−0.04–0.03)	0.00 (0.00–0.00)	0.00 (−0.03–0.04)	0.00 (0.00–0.00)
Pressure injuries	0.06 (−0.02–0.15)	−0.05 (−0.13–0.02)	−0.01 (−0.04–0.02)	0.00 (0.00–0.00)	0.01 (−0.02–0.04)	0.00 (0.00–0.00)
Skin abrasion	**0.16 (0.06–0.26)**	0.06 (−0.1–0.22)	−**0.20 (**−**0.29**–−**0.10)**	−0.03 (−0.08–0.01)	**0.22 (0.12**–**0.33)**	0.03 (−0.01–0.08)
All categories combined	**0.14 (0.06–0.24)**	0.00 (0.00–0.00)	0.00 (0.00–0.00)	0.00 (0.00–0.00)	**0.24 (0.12**–**0.35)**	0.04 (−0.01–0.1)
Gear marks & ecchymosis	**0.28 (0.15–0.38)**	−0.05 (−0.12–0.02)	−0.01 (−0.04–0.01)	0.00 (0.00–0.00)	**0.18 (0.07**–**0.30)**	0.02 (0.00–0.07)
Gear marks & poor exsanguination	**0.32 (0.16–0.44)**	−**0.16 (**−**0.24**–−**0.07)**	0.00 (0.00–0.00)	0.00 (0.00–0.00)	**0.16 (0.05**–**0.28)**	0.02 (0.00–0.06)
Gear marks & pressure injuries	**0.24 (0.09–0.38)**	−0.02 (−0.06–0.01)	0.00 (−0.02–0.00)	0.00 (0.00–0.00)	**0.16 (0.06**–**0.25)**	0.02 (0.00–0.06)
Gear marks & skin abrasion	**0.16 (0.07–0.25)**	−0.05 (−0.16–0.07)	−**0.12 (**−**0.20**–−**0.04)**	−0.02 (−0.05–0.00)	**0.23 (0.13**–**0.33)**	0.04 (0.00–0.09)
Ecchymosis & poor exsanguination	**0.29 (0.19–0.39)**	−**0.11 (**−**0.17**–−**0.04)**	0.04 (0.00–0.02)	0.00 (0.00–0.00)	0.06 (−0.01–0.14)	0.00 (−0.02–0.02)
Ecchymosis & pressure injuries	**0.20 (0.08–0.34)**	−0.01 (−0.04–0.02)	−0.04 (−0.02–0.00)	0.00 (0.00–0.00)	0.06 (0.00–0.12)	0.00 (−0.02–0.02)
Ecchymosis & skin abrasion	**0.16 (0.06–0.26)**	−0.05 (−0.14–0.03)	−0.03 (−0.08–0.00)	0.00 (0.00–0.00)	**0.23 (0.11**–**0.35)**	0.03 (−0.02–0.08)
Poor exsanguination & pressure injuries	**0.28 (0.15–0.40)**	−**0.04 (**−**0.09**–−**0.01)**	0.00 (0.00–0.00)	0.00 (0.00–0.00)	0.01 (−0.04–0.06)	0.00 (0.00–0.00)
Poor exsanguination & skin abrasion	**0.18 (0.06–0.29)**	−0.10 (−0.21–0.01)	0.00 (−0.01–0.01)	0.00 (0.00–0.00)	**0.22 (0.12**–**0.33)**	0.03 (-0.01–0.08)
Pressure injuries & skin abrasion	**0.16 (0.06–0.25)**	0.01 (−0.04–0.05)	−0.01 (−0.03–0.00)	0.00 (0.00–0.00)	**0.22 (0.12**–**0.32)**	0.03 (-0.01–0.08)
Gear marks, ecchymosis & poor exsanguination	**0.29 (0.16–0.41)**	−**0.06 (**−**0.09**–−**0.02)**	0.00 (0.00–0.00)	0.00 (0.00–0.00)	**0.19 (0.07**–**0.33)**	0.02 (0.00–0.07)
Gear marks, ecchymosis & pressure injuries	**0.26 (0.13–0.37)**	0.00 (−0.02–0.00)	0.00 (0.00–0.00)	0.00 (0.00–0.00)	**0.18 (0.07**–**0.30)**	0.02 (0.00–0.07)
Gear marks, ecchymosis & skin abrasion	**0.15 (0.06–0.25)**	−0.04 (−0.11–0.01)	−0.01 (−0.03–0.00)	0.00 (0.00–0.00)	**0.24 (0.13**–**0.35)**	0.04 (−0.01–0.10)
Ecchymosis, poor exsanguination & pressure injuries	**0.29 (0.18–0.40)**	−0.01 (−0.04–0.00)	0.00 (0.00–0.00)	0.00 (0.00–0.00)	0.06 (−0.01–0.14)	0.00 (−0.02–0.00)
Ecchymosis, poor exsanguination & skin abrasion	**0.17 (0.07–0.27)**	−0.05 (−0.10–0.00)	0.00 (0.00–0.00)	0.00(0.00–0.00)	**0.24 (0.11**–**0.37)**	0.03 (−0.02–0.09)
Poor exsanguination, pressure injuries & skin abrasion	**0.17 (0.06–0.28)**	−0.01 (−0.02–0.00)	0.00 (0.00–0.00)	0.00 (0.00–0.00)	**0.22 (0.12**–**0.32)**	0.03 (−0.08–0.08)

The values in black and bold demonstrate a significant difference in the score probability for cod retained in the sequential codend compared with the conventional codend. Non-bold values in black do not prove significant quality differences.

## Discussion

The sequential codend concept significantly improved fish quality compared with a conventional codend. Cod without catch damage were five times as prevalent in the sequential codend (18% had no catch damage) compared with catches from the conventional codend (3.6% had no catch damages). Cod retained within the sequential codend had significantly lower probabilities of incurring gear marks, ecchymosis, poor exsanguination, and skin abrasions. Also the combined scores demonstrated improved quality with the dual sequential codend. The combined scores are relevant as in many cases it is the total amount of damages that results in good or bad quality, as well as the accumulative probabilities for not scoring above a given level. Catch quality determines the applicability of fish for various products, as well as their shelf life [5, 6], e.g. improved exsanguination increases shelf life, fillet whiteness and thus the applicability for high quality demanding products such as fresh fillet loins, and clipfish [1, 9, 11, 15]. In general, low quality fish with severe catch damages, such as poor exsanguination, ecchymosis, gear marks, pressure injuries or skin abrasions are deficient for products requiring whole fish or fillets, and are thus often used in products requiring minced fish, and vice versa. An improvement in catch quality will enable the bottom trawl fishing industry to expand their markets, besides the most common production, i.e. frozen headed and gutted. Thus, the improvements seen in the catch caught using the sequential codend could positively impact fish prices and, therefore, economic returns.

From a management perspective, good catch quality reduces the risk of discarding and high-grading fish [7]. Furthermore, size selection of cod during haul-back, buffer towing or at the surface also increases the likelihood of unaccounted fishing mortality [27]. This results from catch-related damage, such as stress [28, 29] and barotrauma [30–32]. The small-meshed codend, which improved the quality of fish caught, could also reduce the unaccounted mortality, because one can assume that no fish are able to escape when retained in the new codend.

The experimental design enabled inference of any potential difference in the fish quality between the two codend designs, as the two codend were fished simultaneously, minimizing the within haul variance such as catch volume, fish length, towing depth, towing time, weather conditions and season, as well as other possible influencing conditions. Any within haul variance as well as the between haul variance is accounted for by the double bootstrap method applied in the analysis.

The concept of the sequential codend worked as intended. Fish were released into the quality-improving codend segment by untying a choking strop with a release mechanism during haul-back. The inverse hydrostatic catch release mechanism was mounted on the transition between the size-selective codend and the quality-improving codend and was easy to attach and operate before deploying the trawl.

Two enhancements are proposed to further improve the system presented in this study: (i) reducing the mesh sizes in the quality-improving codend segment; and (ii) opening the quality-improving codend segment earlier. Reducing the mesh size in the quality-improving codend segment or replacing some of the codend netting with a tarpaulin-like material could contribute to an even larger improvement in catch quality. To minimize the effect of gravity on the fish, and the crushing weight of the surrounding catch, the codend segment should retain as much water as possible, both during haul-back and when pulled up the slip, without causing a flood when arriving on deck. The codend tested with 6-mm mesh size was observed to retain too little water, because most of the water drained out while the codend was pulled up the slip. The catch releaser opened at a depth of 110 m, which is less than half of the ~250 m fishing depth. Even though this was below the critical 30% limit of the catching depth where the swimbladders of cod rupture [33–35], rapid decompression during the haul-back causes the swimbladder to expand while ascending. The expansion of the swimbladder increases the packing of the fish in the codend, especially if the catches are large. Therefore, catch quality could be increased even more with the sequential codend if the catch releaser was opened at a depth equivalent to, for instance 80%, of the fishing depth. In addition to the suggestions for improvements, further studies should investigate differences in size selectivity between the conventional and sequential codends.

Many studies have documented the importance of correct procedures for processing caught fish to achieve good catch quality [3, 36, 37]. However, with the current processing techniques on board factory trawlers, it is impossible to improve the quality of the catch once this has deteriorated during the catching process. Therefore, preventing fish damage is key to improved fish quality and increased yield from the fisheries. The dual sequential codend concept with a quality-improving codend segment provides a simple method to significantly improve catch quality. It reduces the frequency and severity of catch-related damages such as gear marks, poor exsanguination, ecchymosis and skin abrasion, encountered in a regular trawl codend.
